# Targeting Epigenetics and Non-coding RNAs in Myocardial Infarction: From Mechanisms to Therapeutics

**DOI:** 10.3389/fgene.2021.780649

**Published:** 2021-12-20

**Authors:** Jinhong Chen, Zhichao Liu, Li Ma, Shengwei Gao, Huanjie Fu, Can Wang, Anmin Lu, Baohe Wang, Xufang Gu

**Affiliations:** ^1^ Department of TCM, Tianjin University of TCM, Tianjin, China; ^2^ Acupuncture Department, The First Affiliated Hospital of Tianjin University of TCM, Tianjin, China; ^3^ Department of Cardiology, The Second Affiliated Hospital of Tianjin University of TCM, Tianjin, China

**Keywords:** epigenetics, DNA methylation DNA, histone modifications, non-coding RNAs rna, micro-RNA, cardiovascular, myocardial infarction

## Abstract

Myocardial infarction (MI) is a complicated pathology triggered by numerous environmental and genetic factors. Understanding the effect of epigenetic regulation mechanisms on the cardiovascular disease would advance the field and promote prophylactic methods targeting epigenetic mechanisms. Genetic screening guides individualised MI therapies and surveillance. The present review reported the latest development on the epigenetic regulation of MI in terms of DNA methylation, histone modifications, and microRNA-dependent MI mechanisms and the novel therapies based on epigenetics.

## 1 Introduction

Myocardial infarction (MI) is a common cause of global morbidity and mortality and has caused nearly half of all deaths across Europe, more than 24 million deaths in the United States, and approximately 7–18% of the global 1-year mortality (Nichols et al., 2014; Reed et al., 2017). Atherosclerosis develops in the younger age group and persists for several decades, resulting in MI or other lethal cardiovascular diseases such as heart failure, stroke, and sudden death (Weintraub et al., 2011). Lifestyles changes and effective therapeutic strategies such as diet, abstinence from cigarettes and alcohol, percutaneous coronary intervention, and coronary artery bypass graft resulted in a considerable reduction in MI-induced mortality (Cokkinos and Pantos, 2007; Nichols et al., 2014; Xue et al., 2021). Although patients with MI increased, the life expectancy of patients is almost unaffected (Shibata et al., 2015; Thygesen et al., 2018). A New England Journal of Medicine study reported no evidence comparing an initial invasive strategy to an initial conservative approach to decrease cardiovascular risk or deaths in a median of 3.2 years (Maron et al., 2020). Thus, finding novel biomarkers is necessary for early MI detection. Patients with coronary artery disease (CAD) or MI repeatedly exhibited positive family history (Mayer et al., 2007). Familial genetic defects with an autosomal dominant form resulted in MI in humans (Wang et al., 2003). MI is a complex disease involving both environmental and genetic factors and their interactions. Genetic polymorphisms for numerous genes through atherosclerosis, inflammation and thrombogenesis pathways may account for the susceptibility to MI and severe CAD consequences (Chen et al., 2007). With improved resequencing technology, the gene identification and confirmation methodology can be used for reference in CAD. This enables researchers to better quantify CAD risk in early life, formulate more efficient therapeutic approaches, and reduce the individual probability of developing MI (Damani and Topol, 2007). Genetic linkage studies were performed in both human and animal models to identify these gene polymorphisms. Several genetic association studies have revealed numerous genes and biological pathways (Newton-Cheh and O’Donnell, 2004).

Research work on epigenetics and epigenomics in this field has made remarkable progress and attracted numerous geneticists, molecular biologists, oncologists, and cardiologists. Advancements in epigenetic areas have offered a fresh perspective on human diseases and ushered a new era in genomics by exploring the role of environmental interaction and genetic heritability in disease pathology (Portela and Esteller, 2010; Cao et al., 2014). The heritability of cardiovascular diseases such as myocardial infarction can vary depending on sex, age, and environmental and lifestyle conditions (Saban et al., 2014; Dorn and Matkovich, 2015; Sen et al., 2016; Cavalli and Heard, 2019; Asllanaj et al., 2020; Deegan et al., 2021). Epigenetics is currently a popular biological research area. The term “epigenetics” generally suggests all genetic variations of gene expression regulation except nucleotide sequence and chromatin organisation depending on DNA sequences (Egger et al., 2004; Abi Khalil, 2014). Thus, epigenetic mechanisms related to gene expression regulation are chromatin-based and not involving any DNA sequence changes (Cao et al., 2014). Epigenetic inheritance is a critical mechanism that maintains the dynamic and stable propagation of gene activity states from cells of the last generation to those of the following generation (Kim, 2013; Abi Khalil, 2014).

**FIGURE 1:**
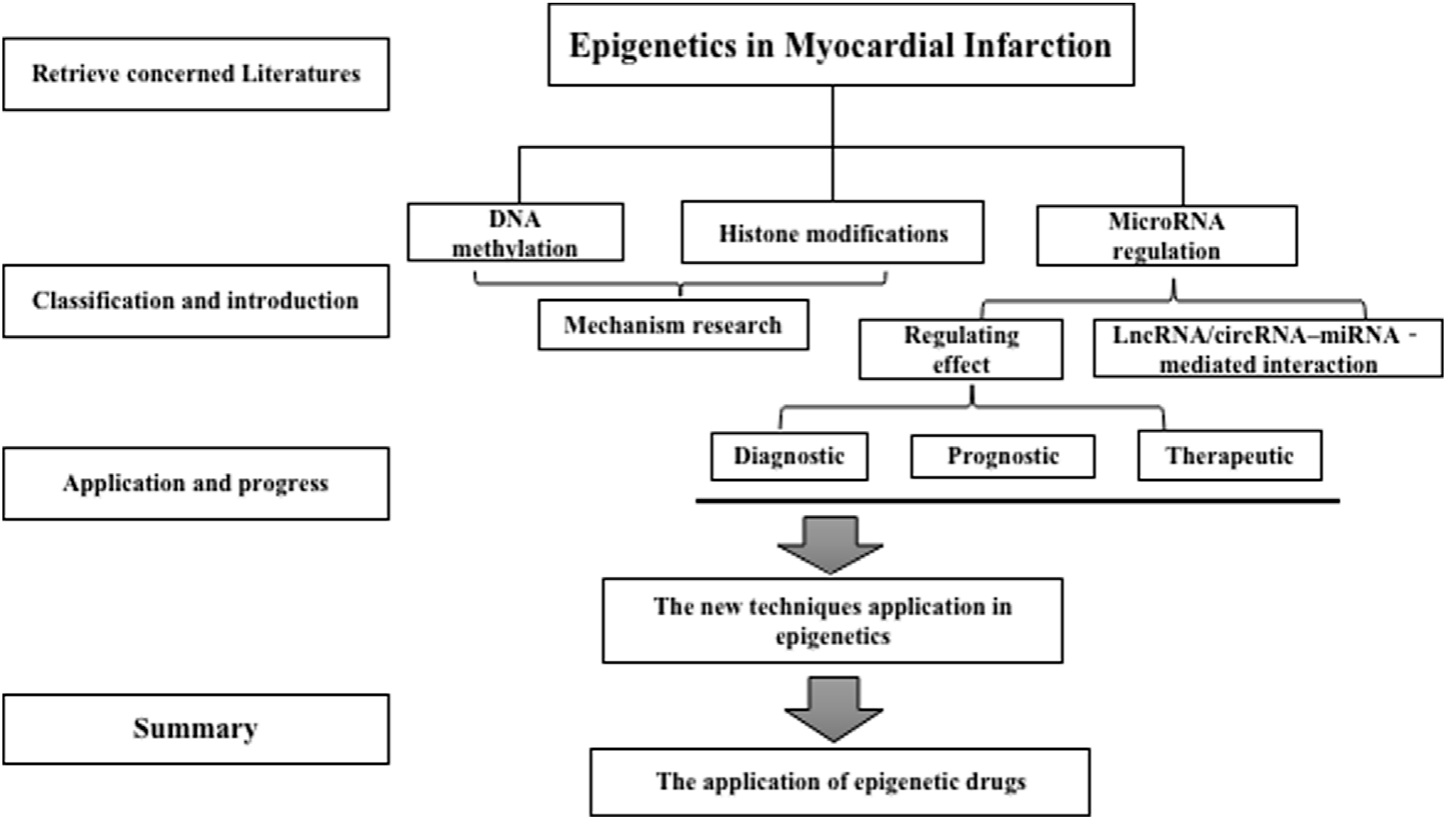
Flow chart.

Epigenetic regulatory processes encompass diverse molecular mechanisms such as DNA methylation (DNAm), histone post-translational modifications, and RNA-based mechanisms such as long non-coding RNAs, lncRNAs, and microRNAs (Kim et al., 2009). In several cases, the epigenetic changes reflect responses to environmental and lifestyle factors, resulting in persistent dynamic changes in gene expression that affect the course of cardiovascular disease. Epigenetic regulators have been increasingly targeted in cancer therapeutics. Thus, epigenetic regulatory mechanisms for cancer and CAD must be explored and are significant in the oncology and cardiology fields (Ding et al., 2018). The epigenome expression can fundamentally differ from different cell types, possibly modulating single cell gene expression by organising nuclear architecture in chromosomes, suppressing or promoting transcription factor access to DNA, and regulating gene expression (Wang and Chang, 2018). Epigenetics dysregulation is considered the cause of many human disorders, such as severe cardiovascular diseases, due to the significance of differential gene regulation in cellular differentiation and application function (Abi Khalil, 2014; Yang et al., 2015; Prasher et al., 2020). Through the search of PubMed, we have summarized a large amount of literature related to epigenetics, aiming to gain insight into their potential application in MI (In Figure 1 -Flow Chart). The present review focused on the crucial role of epigenetic regulatory mechanisms in MI.

## 2 DNA Methylation and MI

Normal DNA methylation, among the central mechanisms regulating gene expression, can decide a severe cardiovascular event (Nurnberg et al., 2020). However, aberrant DNAm with genome-wide hypomethylation and CpG island hypermethylation is also observed in CAD (Breton et al., 2014; Sharma et al., 2014). The development of epigenetic epidemiology increases the probability to investigate the correlations of genomic coding, modifiable exposures, and disease phenotype manifestations. As a vital epigenetic modification type, DNAm plays a significant role as a potential mechanism of such correlations (Zhong et al., 2016). DNA methylation represents a pre-transcriptional modification that can alter the transcriptional process by adding methyl groups onto specific DNA nucleotides (Ma et al., 2014). The process leads to inactive gene expression because the methyl binding protein binds transcriptional factors and DNA. Hypomethylation is more common in most diseases than hypermethylation (Movassagh et al., 2011). DNAm, together with genetic mechanisms, is crucial for natural evolution and maintenance under specific gene expression patterns among mammals. Simultaneously, it is also dynamic and reversible for the regulating effect of genetic mechanisms (Fardi et al., 2018). DNAm pattern changes and the resulting differentially methylated regions have focused on numerous studies on normal development and disease (Ziller et al., 2013). Several studies report the value of epigenetic processes as disease biomarkers, with multiple studies associating DNAm with clinical events (Locke et al., 2019).

DNAm is the most promising target for accurate diagnosis, prognosis, and treatment (Koch et al., 2018). A lack of DNAm markers has been successfully translated into clinical applications. However, the recent improvements in DNA sequencing and other molecular biomedical science technologies result in DNA methylation-based biomarkers exhibiting colossal potential for transforming the treatment and observation of diseases like MI and cancer (Gallardo-Gómez et al., 2018). Several reports reported the role of DNAm in regulating cardiovascular risk factors and myocardial protection in MI, particularly those related to lipid metabolism and myocardial protection against ischaemia (for the detailed description, Table 1). Talens et al. (2012) reported that the risk and developmental components of MI in women are linked to DNA methylation marks at specific loci that were earlier sensitive to prenatal conditions. Aldehyde dehydrogenase 2 (ALDH2) is crucial for protection against myocardial ischaemia. Regulatory T (Treg) cells have been shown to play a protective role in experimental atherosclerosis. Demethylation of the DNA encoding the transcription factor forkhead box P3 (FOXP3) was found to be essential for the stable maintenance of the suppressive properties of Tregs. Lei et al. (Jia et al., 2013)demonstrated that reduction in Treg cells is associated with ACS in atherosclerotic patients. Epigenetic suppression of FOXP3 might lead to down-regulation of Treg cells, and in turn increase the risk of ACS. The Notch pathway plays a key role in stimulating mammalian cardiomyocyte proliferation during development and in the early postnatal life; Analysis of Notch-responsive promoters in adult cardiomyocytes showed marks of repressed chromatin and irreversible CpG DNA methylation (Felician et al., 2014). Wang et al. (2015) proved the correlation between aberrant hypermethylation at CpG sites in animal models in ALDH2 promoter upstream sequence and myocardial ischaemia injury that possibly lead to ALDH2 downregulation after MI. The modulative effects of DNAm on cardiac function, carcinogenesis, and recovery after ischaemic injury, thrombosis, and altered endothelial function in patients with MI have also been investigated. DNA methylation significantly changes following MI. The gene expression proves the correlation of cardiac injury-related epigenetic changes with branched-chain amino acid metabolism (Ward-Caviness et al., 2018). Mathias et al. (Rask-Andersen et al., 2016) observed more than a hundred significant genes for MI pathogenesis or recovery. Similarly, Farzana et al. (Yousuf et al., 2020) reported that hypermethylation of the ABO gene promoter seemingly increases the AMI risk in the hospitalised MI population. Otherwise, gestational diabetes mellitus induced offspring cardiac oxidative stress and DNA hypermethylation, resulting in an epigenetic down-regulation of Sirt1 gene and aberrant development of heart ischemia-sensitive phenotype, which suggests that Sirt 1-mediated signaling is the potential therapeutic target for the heart ischemic disease in offspring (Chen et al., 2019). Not only that, Diabetes increases the vulnerability of STEMI patients to post-MI HF by down-regulating SLN promoter methylation, which further regulates SERCA2a activity via increasing cardiac SLN expression (Liu Z. et al., 2020). Interleukin-6 (IL-6) is implicated in the pathogenesis of coronary heart disease, and IL-6 expression has associated with the level of DNA methylation of its gene promoter. There are two findings suggest that an increase in IL-6 gene expression and its DNA hypomethylation promoter are associated with acute myocardial infarction and CABG surgery patients (Zuo et al., 2016; Mohammadpanah et al., 2020).

**TABLE 1:** The characteristic of DNA methylation in myocardial infarction.

Author	Year	Nation	Detection method	Species	Gene	Site	MI related functional consequences
Talens et al. (2012)	2012	Netherlands	Mass spectrometry	Human	INS、GNASAS	Unknown	Reflected a developmental component of MI.
Jia et al. (2013)	2013	China	PCR	Human	FOXP3	Unknown	Increase the risk of ACS
Felician et al. (2014)	2014	Italy	Bisulfite sequencing	Mouse	Notch	Unknown	Expanded the proliferative capacity of neonatal cardiomyocytes
Wang et al. (2015)	2015	China	Bisulfite sequencing PCR (BSP)	Mouse	ALDH2	Unknown	Myocardial protection against ischaemia
Guarrera et al. (2015)	2015	Italy	Microarray analysis	Human	ZBTB12	Unknown	Angiogenesis and vascular permeability
Rask-Andersen et al. (2016)	2016	Sweden	Infinium humanmethylation450 beadchip	Human	196 genes	211 CpG-sites	Cardiac function, cardiovascular disease, cardiogenesis and recovery after ischaemic injury
Zuo et al. (2016)	2016	China	Bisulfite pyrosequencing	Human	IL-6	3 CpG-sites	DNA hypomethylation of IL-6 promoter is associated with the increased risk for CHD
Mohammadpanah et al. (2020)	2020	Iran					
Nakatochi et al. (2017)	2017	Japan	Infinium humanmethylation450 beadchip	Human	ZFHX3	cg06642177, cg07786668, cg17218495	FHX3 belongs to a susceptibility gene for CVD; SMARCA4 is able to affect inhibition of vascular smooth muscle cell proliferation by hydrogen sulfide
					SMARCA4		
Ward-Caviness et al. (2018)	2018	US	Illumina humanHT-12v3 array	Human	LRP8、KCNN1	9 CpG-sites	Risk factor for MI; reduce ventricular fibrillation and ventricular tachycardia during induced acute myocardial infarction
Agha et al. (2019)	2019	US	Illumina infinium 450k microarray	Human	ATP2B2, CASR, GUCA1B, HPCAL1, CASR, PTPRN2, CDH23, HPCAL1	52 CpG-sites	Calcium regulation
							Serum calcium and serum calcium-related risk of CHD
							Coronary artery calcified plaque
							Kidney function
Chen et al. (2019)	2019	United States	5-mC DNA ELISA	Mouse	Sirt 1	Unknown	Sirt 1-mediated signaling is the potential therapeutic target for the heart ischemic disease in offspring
Koseler et al. (2020)	2020	Turkey	Illumina hiSeq4000	Human	LDAH, APOB, ACSM2A, ACSM5, ACSF3, CES1, CES1P1, AFG3L2, ISCU, SEC14L2, MTTP	Unknown	Cholesterol and lipoprotein metabolism
Yousuf et al. (2020)	2020	Pakistan	Methylation-specific polymerase chain reaction	Human	ABO	Unknown	Thrombosis and altered endothelial function
Fernández-Sanlés et al. (2021)	2021	Spain	Infinium methylationEPIC beadchip	Human	AHRR, PTCD2, intergenic, MPO	cg05575921cg25769469	The four identified CpGs as predictive biomarkers
						cg21566642cg04988978	1Smoking, lipid metabolism, and inflammation

Studies have revealed the complementary expression patterns of lipid metabolism, calcium regulation, and methylation of related genes in peripheral blood leucocyte samples of patients with MI (Agha et al., 2019). These CpGs sites and genes stress the correlation of ion regulation, lipid metabolism, and inflammation in the MI biological mechanisms (Fernández-Sanlés et al., 2021). Thus, the new DNA methylation sequencing technology can identify potential target sites related to the aberrant epigenetic regulation of MI (Koseler et al., 2020). Additionally, the sites stated by two studies (Guarrera et al., 2015; Nakatochi et al., 2017) are candidates for further assessment as underlying MI biomarkers. These results exhibited that DNA methylation could be used as a major molecular process linking genetic variations to MI susceptibility.

## 3 Histone Modifications and HDACs in MI

Histone modification is the primary mechanism in epigenetic regulation, including post-transcriptional modifications, and the most common modifications are phosphorylation, acetylation, methylation, and ubiquitination (Tingare et al., 2013). Such post-transcriptional modifications exert vital biological functions on multiple cellular processes such as cell cycle and metabolism control, DNA repair, and gene transcription (Tang and Zhuang, 2019). Histone deacetylases (HDACs) belonging to transcriptional regulators can serve as a post-translational modifier with different cardiac pathophysiology roles. The basic experiment exhibited that HDAC inhibitors benefit against arrhythmia, MI, cardiac remodelling, hypertension, and fibrosis (Eom and Kook, 2014). Additionally, HDACs are strongly associated with other vascular disorders such as neointima formation, atherosclerosis, and vascular calcification (McKinsey, 2011). Zhang L. et al. (2018) reported acute HDAC effects as positive and negative regulators for pathological cardiac remodelling. Wang J. et al. (2020) uncovered the histone modification profile in the early stage of MI of mice and proved that the modulation of histone modifications could involve inflammation and angiogenesis through adjusting promoters and super enhancers and joining cardiac remodelling pathological processes.

Additionally, the protective effect and therapeutic potential of HDACs were verified by cardiac disease pathogenesis, including suppressing cardiac fibrosis; enhancing angiogenesis; preventing electrical remodelling; and regulating apoptosis, autophagy, and cell cycle arrest (Chun, 2020). Studies exhibited that HDAC enzyme suppression has become a potential candidate for decreasing reperfusion impairment (Xie et al., 2019). Ting et al. (Zhao et al., 2007) reported the use of trichostatin A (TSA) as an efficient HDAC inhibitor to imitate early pharmacologic preconditioning. TSA significantly improved post-ischaemic ventricular function recovery and reduced infarct size during early and delayed preconditioning.

The vital role of HDACs in CVD was greatly emphasized in the past. However, few studies have focused on the association between MI and HDACs (Chen X. et al., 2020). Several studies described the effect of HDACs on vascular dysfunction and MI. Although HDAC could prevent the pathological process of MI in most cases, some HDACs might exacerbate it. Thus, the present study summarised the mechanism and treatment with HDACs, discussed the use of available medicine, and suggested a direction for future clinical studies. The fundamental mechanisms of HDAC action include induction of cardiomyocyte autophagy, augmentation of cardiac remodelling, enhancement of myocardial repairs, and improvement of myocardial ischaemic injury. HDAC inhibitor prevented post-MI cardiac remodelling and depended upon the recovery of autophagosome processing for cardiac fibroblasts. Both clinical trials and animal studies indicated that the HDAC inhibitor TSA could reverse hypoxia-induced impaired autophagic flux and resulted in a 40% reduction in cell death (Wang Y. et al., 2018). Another anticancer, HDAC inhibitor SAHA decreased the myocardial infarct size in an animal model by autophagic flux induction (Xie et al., 2014). HDAC suppression facilitated cardiac repairs and neovascularisation of the infarcted myocardium. Zhang et al. (2012a) proved that c-kit + cardiac stem cell (CSC) preconditioning through HDAC inhibition with trichostatin could substantially increase c-kit + CSC-derived myocytes and microvessels and reinforce *in vivo* functional recovery of MI. However, it is still unclear if specific HDAC4 suppression can modulate CSCs to promote myocardial repair and maintain cardiac performance. HDAC inhibition facilitated c-kit + CSCs to be differentiated into cardiac lineage commitments *in vitro*, whereas HDAC4 overexpression weakened c-kit + CSC-derived cardiogenesis (Zhang et al., 2014).

Additionally, some studies reported that gut microbiota possibly affected the post-MI acetylation levels and tissue repair by influencing butyric acid production (Song et al., 2021). These results prove the role of HDAC4 inhibition in promoting CSC-derived cardiac regeneration and improving cardiac function recovery. Zhang et al. (2018c) demonstrated for the first time that transgenic HDAC overexpression is crucial for the regulation of cardiac function and remodelling.

Although HDAC activation could serve as a regulator of cardiac function in MI, activated HDAC overexpression augmented remodelling. Santhosh et al. (Mani et al., 2015) proved that HDAC inhibition could stimulate myogenesis and angiogenesis under an incubated embryonic stem cell model. HDAC inhibition prevents cardiac remodelling through the stimulation of endogenous regeneration. Additionally, HDAC inhibition improved post-MI myocardial functional recovery through the prevention of myocardial remodelling and a decrease in myocardial and serum tumour necrosis factor α (Zhang et al., 2012b). Thus, HDAC inhibition maintains cardiac performance and relieves myocardial remodelling through the simulation of endogenous cardiac regeneration. Lin et al. (2020) proved that HDAC inhibition could stimulate proteasome-dependent degradation of HDAC4, which may be related to HDAC4 sumoylation to provoke such protective effects. Du et al. (2015) discovered that HDAC inhibition avoids cell death, promotes cell-viability, and decreases ROS production and apoptosis of cardiomyocytes under exposure to H/R. These studies offer a novel understanding of the molecular mechanism of HDAC inhibition and the potential development of specific HDAC inhibitors as new MI therapies.

## 4 Non-coding RNAs and MI

Although more than 90% of human genomes cannot encode proteins, they exhibit high transcriptional activity and generate a broad spectrum for non-coding RNAs having regulatory and structural functions (Mattick et al., 2010). MicroRNAs (miRNAs), small interference RNAs (siRNAs), long non-coding RNAs (lncRNAs), and circular RNAs (circRNAs) exert regulatory functions or diagnostic potential against CVDs (Poller et al., 2018). All non-coding RNAs are MI biomarkers (Wang and Jing, 2018). The present study also analysed these ncRNAs and associated interactions in regulating cardiomyocyte apoptosis, inflammation, angiogenesis, and fibrosis following the acute setting to understand their potential in acute MI treatment (Guo Y. et al., 2017; Poller et al., 2018). The present review summarises the latest advances and future applications for non-coding RNAs as MI biomarkers and focuses on the diagnostic value, prognostic potential and therapeutic effect in such RNAs. Several animals and clinical studies demonstrated the diagnostic value, prognostic potential and therapeutic effect for MI associated miRNAs.

### 4.1 miRNAs and MI

#### 4.1.1 miRNAs as Diagnostic Biomarkers of MI

Despite the difference in sensitivity and accuracy among circulating miRNAs, some new circulating miRNAs containing unique release kinetics can be used as promising candidates for acute MI diagnostic biomarkers. Most of the circulating miRNAs could function as diagnostic biomarkers of acute MI (In Table2). In AMI patients, the upper levels of miR-19a (Mansouri and Seyed Mohammadzad, 2020), miR-22-5p,miR-122-5p (Wang Y. et al., 2019), miR-23b (Zhang J. et al., 2018), miR93-5p (O Sullivan et al., 2016), miRNA-124 (Guo ML. et al., 2017), miR-134-5p, miR-186-5p (Wang et al., 2016), miR-139-5p (Wang C. et al., 2021), miR-181a (Zhu et al., 2016),miR-208b, miR-499 (Agiannitopoulos et al., 2018),miR-328, miR-492 (Guo LL. et al., 2020), miR-1291and miR-663b (Peng et al., 2014) were significantly correlated with the increased serum levels of CK-MB and cTnI. On the contrary, the level of miR-99a (Yang SY. et al., 2016), miR-379 (Yi and An, 2018), miR6718 and miR-4329 (Chen S. et al., 2021) had a negative correlation with cTnI level and CK-MB in the AMI patients. Besides, miR-139-5p inhibited endothelial cell viability of AMI by inhibiting VEGFR-1, and increased miR-139-5p expression in AMI patients has high diagnostic value for AMI screening (Wang C. et al., 2021). Correlation analysis showed that plasma miR-181a was positively correlated with coronary Gensini score and negatively correlated with left ventricular ejection fraction. Relative miR-181a levels in AMI patients were positively correlated with the concentrations of the creatine kinase-MB fraction and cardiac troponin I (Zhu et al., 2016). The over-expression of miR-208a in myocardial infarction tissue and the high levels of this miRNA in the serum, may be involved in the process of myocardial infarction by influencing the cAMP-PKA signaling pathway in myocardial cells (Feng et al., 2016). At the same time, some circulating miRNAs were used for ischaemic risk stratification (He et al., 2017; Hromadka et al., 2021) prediction of the major adverse cardiovascular events after AMI future occurrence rate of MACE (Liu et al., 2017; Guo X. et al., 2020) or prognostic value of left ventricular (LV) dysfunction and symptoms of heart failure following acute MI (Maciejak et al., 2018). These results verified that constructing a complete network for circulating miRNAs after MI allows rapid MI diagnosis and opens novel therapeutic opportunities of MI, thus providing personalised therapies for patients at MI risk (Wang and Jing, 2018).

**TABLE 2:** The diagnostic value of MI associated miRNAs.

Author	NcRNAs	Research types	Clinical value
Mansouri and Seyed Mohammadzad (2020)	miR-19a	Clinical research	The upper levels of miR-19a were significantly correlated with the increased serum levels of CK-MB, CTn I and creatinine
Wang et al. (2019b)	miR-22-5p, miR-122-5p	Clinical research	Plasma miR-122-5p levels is significantly elevated in AMI patients, while plasma miR-22-5p levels were significantly decreased. In addition, significant correlations between miR-22-5p and miR-122-5p, miR-122-5p and creatine kinase isoenzyme were detected
Zhang et al. (2018a)	miR-23b	Clinical research	Circulating miR-23b as a novel biomarker for early risk stratification after ST-elevation myocardial infarction
O Sullivan et al. (2016)	miR-93-5p	Clinical research	It was the strongest predictor for CAD following the adjustment of conventional risk factors, showing underlying diagnostic utility
Yang et al. (2016b)	miR-99a	Clinical research	The expression of miR-99a was significantly downregulated in patients with AMI. In the AMI patients, miR-99a level had a negative correlation with cTnI level and CK-MB.
Guo et al. (2017a)	miR-124	Clinical research	MiRNA-124 expression in experimental group was significantly elevated in peripheral blood of AMI patients
Wang et al. (2016)	miR-134-5p	Clinical research	Levels of plasma miR-19b-3p, miR-134-5p and miR-186-5p were significantly increased in early stage of AMI. In addition, all three miRNAs were positively correlated with cTnI
Wang et al. (2021a)	miR-139-5p	Clinical research	miR-139-5p inhibits endothelial cell viability of AMI by inhibiting VEGFR-1, and increased miR-139-5p expression in AMI patients has high diagnostic value for AMI screening
Zhu et al. (2016)	miR-181a	Clinical research	Relative miR-181a levels in AMI patients were positively correlated with the concentrations of the creatine kinase-MB fraction and cardiac troponin I.And plasma miR-181a was positively correlated with coronary Gensini score and negatively correlated with left ventricular ejection fraction
Feng et al. (2016)	miR-208a	Animal experiment	The over-expression of miR-208a in myocardial infarction tissue and the high levels of this miRNA in the serum, may be involved in the process of myocardial infarction by influencing the cAMP-PKA signaling pathway in myocardial cells
Agiannitopoulos et al. (2018)	miR-208b	Clinical research	miR-208b and miR-499 displayed similar properties with the established AMI biomarker cTnT
	miR-499		
Wang et al. (2011)	miR-328	Clinical research	There was a correlation between circulating miR-133 or miR-328 levels and cardiac troponin I
Yi and An, (2018)	miR-379	Clinical research	Studies demonstrated the miR-379 was negatively correlated with CK-MB and cTns in study subjects.Function assay *in vitro* further indicated miR-379 inhibited cell proliferation and induced cell cycle G0/G1 arrest in VSMCs
Guo et al. (2020a)	miR-492	Clinical research	Serum miRNA-499 and miRNA-210 were associated with MI within 3 h of symptom onset.
Peng et al. (2014)	miR-1291	Clinical research	The levels of miR-133, miR-1291 and miR-663b are associated with AMI.
Chen et al. (2021a)	miR-6718-5p and miR-4329	Clinical research	The expression of miR6718 and miR-4329 in patients with myocardial infarction was significantly lower than that in normal people

#### 4.1.2 The Prognostic Value of MI Associated miRNAs

The prognosis prediction of myocardial infarction is beneficial to delay the progression of heart failure, reduce the mortality of cardiovascular events, and prolong the survival time of patients. We found that mRNAs could be used not only as an independent factor of cardiovascular risk events, but also as a predictor of the development of myocardial infarction (In Table 3). According to the literature, it has been confirmed that miR-1 (Su et al., 2020), miR-30a-5p (Maciejak et al., 2018), miR-223-3p and miR-126-3p (Hromadka et al., 2021) can be used to predict AMI prognosis after MI. Numerous clinical researches in MI patients identified miR-30e (Su et al., 2018), miR-142 (Guo X. et al., 2020), miR-184 (Liu et al., 2017) and miR-221-3p (Coskunpinar et al., 2016), in particular, as the most significantly changing miRNAs in MI, miR-142 and miR-184 over-expression analysis showed that aberrant their levels effect the future occurrence rate of MACE and the function of cardiovascular. Furthermore, miR-145 (Zhang et al., 2017), miR-155 (Zhang B. et al., 2019) and miR-365 (Wu H.-B. et al., 2021) expression also could be used to assess the severity of the patients with HF and prognosticate cardiac function and the risk to develop heart failure.

**TABLE 3:** The prognostic value of MI associated miRNAs.

Author	NcRNAs	Clinical value
Su et al. (2020)	miR-1	miR-1 is an independent risk factor for the prognosis of AMI and can be used to predict AMI prognosis
Maciejak et al. (2018)	miR-30a-5p	miR-30a-5p as a prognostic biomarker of left ventricular dysfunction after acute myocardial infarction
Su et al. (2018)	miR-30e	Association of miRNA-30e with a no-reflow phenomenon in STEMI patients receiving primary coronary intervention
Cortez-Dias et al. (2016)	miR-122-5p/133b	The miR-122-5p/133b ratio is a new prognostic biomarker for the early identification of STEMI patients at a higher risk of developing major adverse events after undergoing PCI intervention
Hromadka et al. (2021)	miR-126-3p	The miR-223-3p and the miR-126-3p are promising independent predictors of thrombotic events and can be used for ischemic risk stratification after AMI.
	miR-223-3p	
Guo et al. (2020b)	miR-142	Predictor of the major adverse cardiovascular and cerebrovascular events (MACCE) in AMI patients
Zhang et al. (2017)	miR-145	Prognosticate cardiac function and the risk to develop heart failure
Zhang et al. (2019a)	miR-155	miR-155 expression could be used to assess the severity of the patients with HF.
Liu et al. (2017)	miR-184	Related to ventricular remodelling indexes and the future occurrence rate of MACE
Coskunpinar et al. (2016)	miR-221-3p	miR-221-3p has a high discriminative value and significant relations with left ventricular systolic function
Horváth et al. (2020)	miR-331	It may be associated with plaque rupture
	miR-151-3p	
Wu et al. (2021a)	miR-365	Heart failure with reduced ejection fraction following myocardial infarction

#### 4.1.3 The Therapeutic Application of MI Associated miRNAs

Cardiac injury was accompanied by dynamic changes in the expression of miRNAs (In Table 4). Related studies have successively reported on the therapeutic effect of MI in patients with myocardial infarction. Li S. et al. (2018) found that downregulation of phosphatase and tensin homolog (PTEN), by the PTEN inhibitor bpV, increased miRNA-23a expression and suppressed the Bax/Bcl-2 protein expression ratio, caspase-3 activity level and p53 protein expression. It indicated that the expression of miRNA-23a may regulate AMI through targeting PTEN in patients and *in vitro*. Studies (Bonauer et al., 2009) have shown that the miR-17approximately92 cluster is highly expressed in human endothelial cells and that miR-92a, a component of this cluster, controls the growth of new blood vessels. Besides, miR-92a appears to target mRNAs corresponding to several proangiogenic proteins, including the integrin subunit alpha5. It may serve as a valuable therapeutic target in the setting of ischemic disease.

**TABLE 4:** The therapeutic application of MI associated miRNAs.

Author	NcRNAs	Research types	Therapeutic action
Li et al. (2018b)	miR-23a	Clinical research	The expression of miRNA-23a may regulate AMI through targeting PTEN in patients and *in vitro*
Zhu et al. (2021b)	miR-26b	Clinical research	A novel therapeutic target of MI
Bonauer et al. (2009)	miR-92a	Animal experiment	As a valuable therapeutic target in the setting of ischaemic disease
Xiang and Yang, (2020)	miR-135b	Clinical research	As a potential therapeutic target in the treatment of MI
Hu et al. (2021)			
Li et al. (2018a)	miR-144	Animal experiment	As a therapeutic agent after MI
Bayoumi et al. (2017)	miR-532	Animal experiment	Be suitable for therapeutic intervention in ischaemic heart disease
Hui et al. (2017)	miR-539	Animal experiment	Possibly a potential therapeutic target for myocardial infarction

In animal and cell experiments, there are three miRNAs have been shown to potentially treat MI, including miR-144, miRNA-532 and miR-539 (Bayoumi et al., 2017; Hui et al., 2017; Li J. et al., 2018). Interestingly, miR-144 provides potent acute cardioprotection in an ischemia/reperfusion injury model and Intravenous miR-144 has potent effects on post-MI remodeling. MiRNA-532 protects the heart in acute myocardial infarction, and represses prss23, a positive regulator of endothelial-to-mesenchymal transition. Overexpression of miR-539 plays a role in the degree of myocardial infarction. The results of experiments demonstrated an increase in the expression of miR-539 and a decrease in the expression of MEK, which led not only to suppressed proliferation but also to apoptosis and autophagy of H9C2 cells. Although other miRNAs also have been proposed to have anti-myocardial infarction effects, it still needs further experimental verification (Xiang and Yang, 2020; Zhu et al., 2021b; Hu et al., 2021).

### 4.2 Regulation of Fibrosis in Infarct Regions by MiRNAs

**FIGURE 2:**
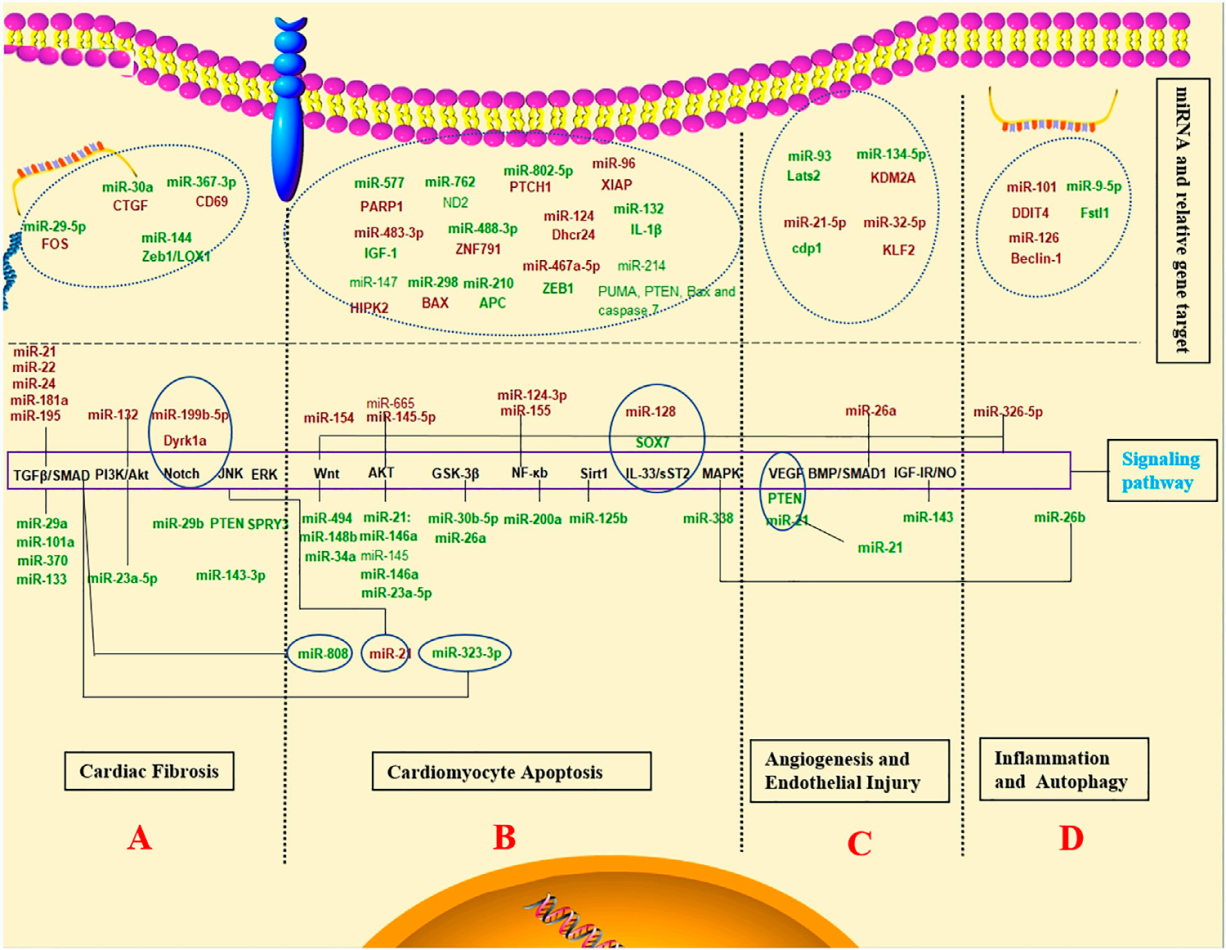
The diagnostic value, prognostic potential and therapeutic effect for MI associated miRNAs. Schematic representation of the activated pathway program in MI. The regulation mechanism of miRNAs networks in MI. (Red and green labels correspond with induced or repressed molecules in MI, respectively).

Major processes leading to post-infarction injury and following remodelling responses are controlled by miRNAs. For example, miRNAs may assist or prohibit cardiomyocyte cell necrosis, modulate post-ischaemic neovascularisation, and control cardiac fibrosis (In Figure 2A).

Some non-beneficial miRNAs regulate cardiac fibrosis to promote remodelling. miR-21, miR-22, miR-24, miR-133, miR-181a, and miR-195 can be upregulated in response to MI and are involved in cardiac fibrosis by tumour growth factor-β (TGF-β) signalling pathway (Hong et al., 2016; Chen et al., 2017; Yuan et al., 2017, 7; Chen P. et al., 2018; Yu et al., 2019; Wang DM. et al., 2020). On the other hand, miR-29a, miR-101a, and miR-370 inhibition protect against cardiac injury following MI (Xiao et al., 2017; Yuan and Gao, 2017). Similarly, miR-29b-3p degraded the pro-fibrosis effect from TGF-β1 through FOS targeting, which provided a promising therapy for post-MI cardiac fibrosis (Xue et al., 2020). CTGF expression was possibly inhibited by MiR-30a through direct combination with the 3′-UTR site of CTGF following MI, reducing collagen generation in myocardia, inhibiting myocardial fibrosis, and improving cardiac function (Chen L. et al., 2018). Yuan et al. (Yuan et al., 2019) discovered the involvement of miR-144 in extracellular matrix remodelling after MI, in which its loss results in enhanced myocardial fibrosis and damaged functional recovery. In animal experiments, miR-29b and miR-199b-5p have been inhibited myocardial fibrosis and cardiac hypertrophy by activating the Notch signaling pathway and protected myocardium against myocardial infarction (Duygu et al., 2017; Liu et al., 2019). Additionally, miR-143-3p and miR-494 promoted fibrosis through different signalling pathways such as ERK, JNK, and Wnt pathways (Li C. et al., 2019; Su et al., 2019). The regulation of these miRNAs can provide novel therapies for MI.

### 4.3 miRNAs in Cardiomyocyte Apoptosis

The relevant miRNA mechanisms in cardiomyocyte apoptosis are summarised in In Figures 2B. The Wnt/β-catenin and PI3K/AKT pathways constitute two major signalling pathways to inhibit apoptosis, which can be constantly activated by activating the pro-apoptotic pathway following acute MI. Some miRNAs protect cardiomyocytes from apoptosis following acute MI through activation of Wnt/β-catenin and PI3K/AKT pathways and their downstream regulators. MiR-30b-5p participates in myocardial cell proliferation and apoptosis by modulation of the Wnt/β-catenin signalling pathway, possibly providing the new underlying target to diagnose MI in the clinic (Chi et al., 2020). MiR-34a affects myocardial cell apoptosis by regulating the activation and inactivation of the Wnt/β-catenin signalling pathway (Li JH. et al., 2019). The miR-148b inhibition reinforced the antioxidative capacity and myocardial cell survival to inhibit apoptosis by activating the Wnt/β-catenin signalling pathway, improving myocardial I/R injury (Yang et al., 2019). Moreover, miR-154 can activate the Wnt/β-catenin signalling pathway, eventually promoting myocardial apoptosis (Sun HY. et al., 2019). Additionally, miR-23a-5p-PI3K/Akt axis regulated apoptosis in MI. Thus, the new axis was incorporated as an underlying indicator for detecting ischaemic heart disease and therapeutic intervention (Huang J. et al., 2020). Furthermore, the apoptosis-associated protein expression levels rose significantly in H9c2 cells transfected with miR-145-5p mimic. MiR-145-5p may inactivate the PI3K/Akt pathway to assist MI cell apoptosis (Huangfu et al., 2020). Other miRNAs can also promote or inhibit myocardial apoptosis after acute MI via different pathways and relevant targets. The miRNA-21 expression experienced upregulation in the serum of elderly patients with acute MI, which suppressed TNF-a caused apoptosis in HCM through activation of the JNK/p38/caspase-3 signalling pathway (Wang Z.-H. et al., 2017). MiR-26a activates the GSK-3β signalling pathway to inhibit myocardial cell apoptosis after acute MI (Lu and Lu, 2020). MiRNA-145 suppresses myocardial infarction-induced apoptosis through autophagy-related to the Akt3/mTOR signalling pathway *in vivo* and *in vitro* (Yan et al., 2018). MiR-214 and miR-203 have abilities to alleviate MI-caused injury on myocardium tissues and reduce mitochondria-mediated apoptosis, which might be a possible mechanism in protecting against AMI injury (Yang X. et al., 2016; Zhang J. et al., 2019). Exosomal miR-338 can inhibit cardiomyocyte apoptosis and improve cardiac function in rats suffering MI by regulating the MAP3K2/JNK signalling pathway (Fu et al., 2020).

Conversely, parts of miRNAs could promote myocardial apoptosis. MiR-96 facilitated acute MI progression by directly targeting XIAP and suppressing XIAP anti-apoptotic function, providing a new therapeutic target to treat acute MI (Wang J. et al., 2021). A mice model exhibited an increase in MI by miR-467a-5p through ZEB1 expression regulation (Huang et al., 2021). miR-665 downregulation protected from cardiomyocyte ischaemia/reperfusion injury-induced ROS accumulation and apoptosis by activating Pak1/Akt signalling of MI (Liu C. et al., 2020).

#### 4.3.1 MiRNAs in Angiogenesis and Endothelial Injury

Numerous clinical studies have attempted to stimulate angiogenesis to combat ischaemic pathologies and tissue injury. These studies have primarily focused on the intra-arterial introduction of a range of angiogenic growth factors such as VEGF (Yang F. et al., 2016), insulin-like growth factor 1 receptor (IGF) (Geng et al., 2020), and HGF (Fan et al., 2018) to promote neovascularisation and tissue perfusion in subjects with MI. Additionally, Liao et al. (Liao et al., 2021) observed that cardiac telocyte suppressed cardiac microvascular endothelial cell apoptosis by exosomal miRNA-21-5p-targeted Cdip1 silencing to ameliorate angiogenesis of MI. Another study from China demonstrated that miR-134-5p silencing facilitated myocardial angiogenesis and suppressed myocardial apoptosis through KDM2A upregulation in MI mice (Li X. et al., 2020). MiR-93 may promote angiogenesis and weaken remodelling by inactivating the Hippo/Yap pathway through Lats2 targeting (Ma et al., 2020). Endothelial injury is crucial for numerous physiological processes and is closely related to tissue repair and recovery after an injury caused by pathological conditions (Icli et al., 2020). Cellular and molecular mechanisms can assist the formulation of novel cardiac cell therapies for the functional and structural regeneration of impaired myocardium. (In Figure 2C).

#### 4.3.2 MicroRNAs Regulate Inflammation and Autophagy

Autophagy is a well-organised homeostatic cellular process responsible for removing damaged organelles and intracellular pathogens. Furthermore, it can modulate the innate and adaptive immune systems and suppress gene expression by targeting messenger RNAs for translational repression. The present study summarised the regulation of different non-coding RNAs in autophagy and other mechanisms (In Figure 2D). Several studies indicated that miRNAs regulate autophagy through different pathways and exhibit a significant influence on MI treatment. Li Q. et al. (2020) referred to MI attenuated by miR-101 induced injury through targeting DDIT4 to modulate autophagy, which implicated miR-101 or DDIT4 as targets for MI. Likewise, miR-126 downregulation will lead to the overactivation of myocardial autophagy induced by Beclin-1, an autophagy-related protein (Shi et al., 2020). MiR-21 suppresses the inflammatory responses in the early phase of MI through targeting KBTBD7 and attenuating MKK3/6 activation of immune cells, thus avoiding excessive scar formation and improving cardiac function (Yang et al., 2018, 7). One study found that miR-26b alleviates inflammatory response and myocardial remodelling in mice with MI by suppressing the MAPK pathway by binding to PTGS2 (Ge et al., 2019). These inflammatory and autophagy miRNAs might be potent therapeutic targets in the setting of MI.

### 4.4 LncRNA/circRNA–miRNA-Mediated Interaction

The development of human genome sequencing and annotation technologies has indicated that the human genome comprises numerous non-coding lncRNA regions (Castellanos-Rubio and Ghosh, 2019). lncRNAs refer to RNA molecules over 200 bp in length without protein-coding potential (Beermann et al., 2016). Additionally, the new regulatory mechanism for lncRNA/circRNA, miRNA, and mRNA has aroused concerns (Hansen et al., 2013; Schmitz et al., 2016). The interaction of lncRNAs and circRNAs with miRNAs influences related mRNA expression. As we know, lncRNAs and circRNAs both contain complementary binding sites to miRNAs and act as endogenous miRNA sponges; miRNAs in turn interact with mRNAs, serving as negative regulators of protein expression. Therefore, LncRNAs and circRNAs function as molecular regulators by determining gene expression.

**FIGURE 3:**
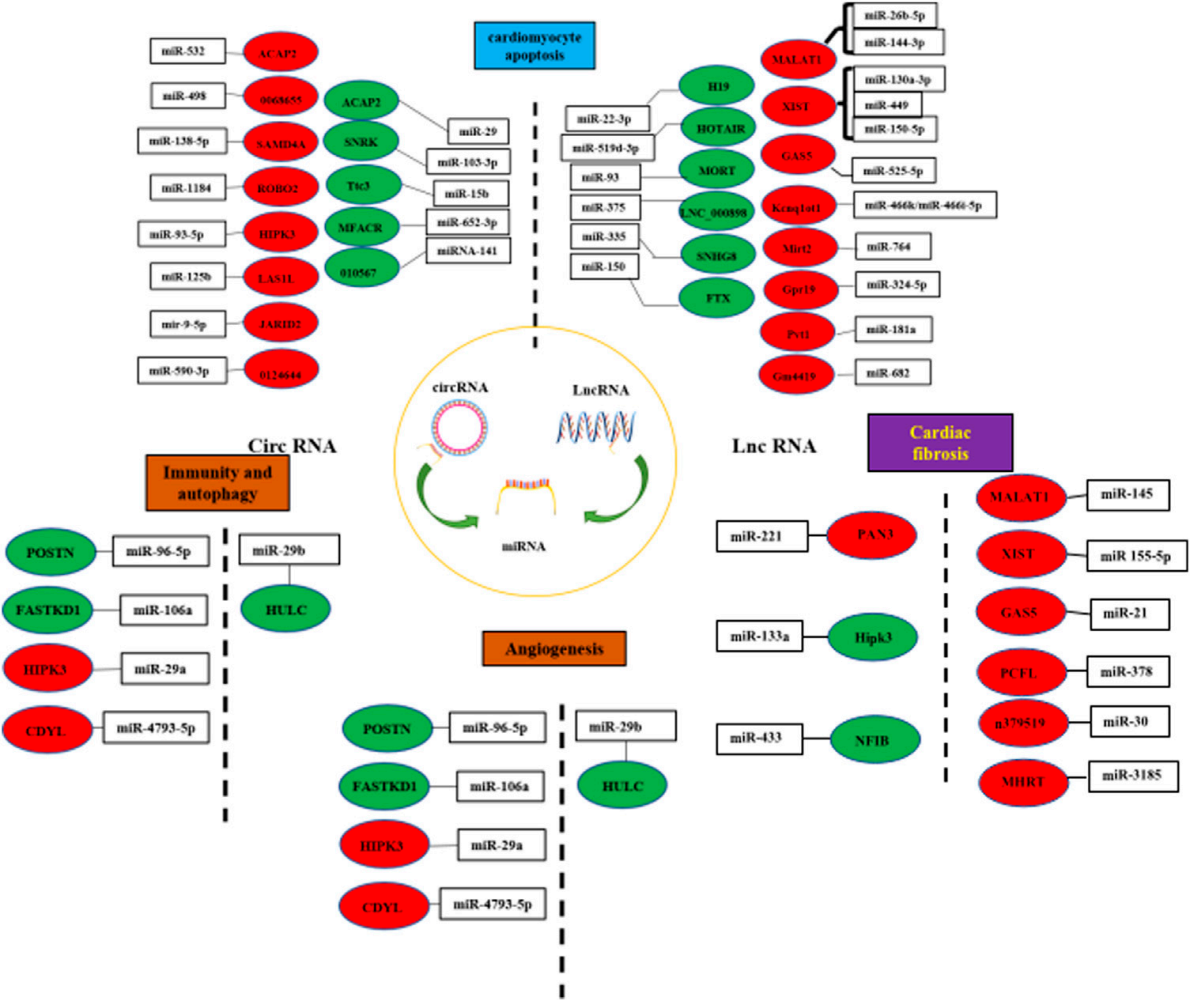
LncRNA/circRNA–miRNA-mediated interaction. (LncRNAs/circRNAs interact with miRNAs to modulate cardiomyocyte apoptosis, cardiac fibrosis, anglognesis, immunity and autophagy. Pro-factor lncRNAs/circRNAs are marked in blue. Anti-factor lncRNAs/circRNAs are marked in red.) (The left side of the dashed line represents circRNA, and the right side represents LncRNA).

lncRNAs take up a large proportion of genes that have differential expression in response to different stress stimuli. After being induced, lncRNAs will regulate downstream cellular processes such as feedback regulation for essential stress response proteins (Valadkhan and Valencia-Hipólito, 2016). Although the significance of lncRNA molecules during various biological processes has been recognised, several details remain unclear. Presently, experiments concerning the functional role of lncRNAs were performed under experimental animal models or by *in vitro* assays. Some studies revealed the active role of lncRNAs in cell autophagy (Liang et al., 2020; Zhang and He, 2020; Li J. et al., 2021), apoptosis (Zhu et al., 2018; Gong et al., 2019; Zhang D. et al., 2019, Zhang M. et al., 2019, Zhang Y. et al., 2019; Zhou et al., 2019; Huang L. et al., 2020; Liao et al., 2020; Lv et al., 2020; Yan et al., 2020; Zhang Y. et al., 2020; Zhou et al., 2020; Chen Y. et al., 2021; Liu et al., 2021; Zhu et al., 2021a, 1), cardiac fibrosis (Wang X. et al., 2018, 30; Huang et al., 2019; Sun F. et al., 2019; Zhang JC. et al., 2019, 21; Lang et al., 2021; Zhang H. et al., 2021, 155–5), cardiac remodelling (Liu B. et al., 2020; Zhang B.f. et al., 2020), inflammation, and angiogenesis (Chen ZL. et al., 2020; Zhao et al., 2020) (In Figure 3).

Further research on the role of lncRNA in MI and cardiovascular events can deepen the understanding of the lncRNA network, contributing to the regulation of gene expression changes underlying MI, and assist the development of specific therapies based on the interference of miRNAs and lncRNA vital to MI.

### 4.5 CircRNAs and MI

CirRNAs are crucial for the physiology and pathology of biological systems and are involved in disease development. CircRNAs refer to a type of non-coding RNAs with higher stability than linear RNAs because they form a covalently closed continual loop with resistance against RNase R activity (Gao et al., 2015). Non-coding RNAs were optimal regulators of the cardiovascular system, and circRNAs were associated with CVDs (Wang W. et al., 2019).

Under component derivation, circRNAs can be usually classified into three categories, namely exon-derived circRNA (ecircRNA), lariat intron-derived circRNA (ciRNA), and exon-derived circRNA with retained introns (EIciRNA) (Li et al., 2015). Most known circRNAs originate from exons (Bei et al., 2018). CircRNAs maintain high stability and are abundantly expressed, making them better biomarkers relative to linear RNAs (Zhou et al., 2018). Simultaneously, with progress in bioinformatics and high-throughput sequencing technology, circRNAs have become a research direction for multiple biological functions and provide novel diagnostic methods and therapies for CVDs (Sun J.-Y. et al., 2020).

The present study addresses the regulatory role and functions of circRNAs, discusses the latest studies, and investigates the role and the regulatory mechanism of circRNAs in MI. Moreover, the roles of circRNAs in multiple MI such as myocardial apoptosis (Wang K. et al., 2017; Cai et al., 2019; Chai et al., 2020; Liu X. et al., 2020, 29; Wang Y. et al., 2020; Zhai et al., 2020; Chen T.-P. et al., 2021; Wu Y. et al., 2021; Zhang J. et al., 2021; Zhao B. et al., 2021; Zhu Y. et al., 2021), autophagy regulation, inflammatory response (Hu et al., 2020; Zhu Y. et al., 2020; Bian et al., 2021; Cai et al., 2021, 3), improvement of fibrosis (Zhu et al., 2019; Li F. et al., 2020; Sun L.y. et al., 2020; Si et al., 2020), and effects of ventricular remodelling (Garikipati et al., 2019; Cheng et al., 2020) (Gao et al., 2020; Zhang M. et al., 2020; Tan et al., 2021, 4; Zhao Q. et al., 2021) have been summarised (Figure 3). circRNAs mediate the fundamental physiological and pathological MI processes. Furthermore, since their dynamic changes can exhibit various disease stages, they are defined as ideal biomarkers. Thus, the present study also summed up the latest development of the role of circRNAs in MI and for convenience of reference.

## 5 The New Techniques and Target Drugs of MI in Epigenetics

The advent of high throughput epigenome mapping technologies has ushered in a new era of multi-omics where powerful tools can now delineate and record different layers of genomic output. Epigenetics play a central role in the regulation of many important biological processes. Despite significant technological advances for epigenetic profiling, there is still a need for a systematic understanding of how epigenetics shapes biological pathway, and disease pathogenesis (Angarica and Del Sol, 2017). DNA methylation and histone modifications and numerous techniques had been invented to analyze epigenetic processes not only at the level of specific genes, but also to analyze epigenetic changes that occur in defined regions of the genome as well as genome-wide. These technologies that are currently driving the field of epigenetics will greatly facilitate continued expansion of this exponentially growing discipline of genetics. A major breakthrough in the analysis of DNA methylation occurred with the development of bisulfite methylation sequencing (Wang and Chang, 2018). It used to be a gold-standard for detection of DNA methylation largely because it allows identification of 5-methylcytosine. This leading method of DNA methylation analysis has led to numerous subsequent methods such as Methylation Specific PCR and so on. Many proteins interact with RNA to modulate RNA-based epigenetic processes. Reaserches usually used the tools available to detect direct and indirect interactions between specific proteins and RNA *in vivo*. This is best achieved through the RNA immunoprecipitation technique (RIP). The uses of the RIP technique are vast and may be applied to epigenetics to help unravel the increasingly appreciated role of RNA in epigenetic processes (Tollefsbol, 2011). Beside, with the rapid development of technology, a number of epigenetic tests have emerged, such as Infinium Methylation450/850 BeadChips (450/850K), Methylated DNA immunoprecipitation-sequencing (MeDIP-Seq), Methylation-specificPCR (MSP),Pyrosequencing; Reduced representation bisulfite sequencing (RRBS), EWAS and so on (Feng and Lou, 2019). Researches should strictly choose appropriate detection methods according to the research direction. The availability of ultra-deep sequencing of genomic will transform the medical in analysis of the causes of disease, development of new drugs and diagnostics fields in the near future (Pareek et al., 2011).

DNA methylation, histone modification, nucleosome remodeling, and RNA-mediated targeting regulate many biological processes that are fundamental to the genesis of cancer. Along with the promising clinical and preclinical results seen with epigenetic drugs against chromatin regulators, signifies that it is the central role of epigenetics in cancer (Dawson and Kouzarides, 2012). Most of the drug research and development carried out from the perspective of epigenetics are related to tumors (Asano, 2020). At the same time, dietary intake has also presented significant influence on human health and disease development and nutritional modifications have proven important in prevention, but also the treatment of disease (Lundstrom, 2019). There are many epigenetic drugs have been identified in the past decade that effectively prevented or treated atherosclerosis and myocardial ischemia in several translational animal models, raising the possibility to combat coronary heart disease by targeting epigenetic processes also in humans. We have summarized several epigenetic therapy agents and strategies that may be associated with myocardial infarction by searching published reviews, including DNMT inhibitors, TET2 activators, Histone deacetylase inhibitors (Pickell et al., 2020), Sirtuin activating compounds, EZH2 inhibitors, BET inhibitor and other target epigenetic processes in atherosclerosis and associated vascular diseases (Schiano et al., 2015; Voelter-Mahlknecht, 2016; Xu et al., 2019). Interestingly, some dietary compounds, including polyphenols, cocoa, and folic acid, can modulate DNA methylation status, whereas statins may promote epigenetic-based control in CVD prevention through histone modifications (Voelter-Mahlknecht, 2016). Unfortunately, according to our knowledge, no epigenetically active agents or drugs targeting histone acetylation and/or methylation have thus far entered clinical trials for MI, nor have any of the latter been approved by the US Food and Drug Administration. The complex relationship between epigenetic regulation and MI development clearly demands further studies (Voelter-Mahlknecht, 2016).

## 6 Conclusion and Perspectives

MI exhibits the maximum morbidity, mortality, and effect on life quality among CVDs worldwide. Considerable progress has been attained in the discovery of MI genetic bases. Though the prospects entailed by understanding and controlling transcription through studies on histone and DNA modifications has received extensive attention, the reading of histone marks once placed shouldn’t been ignored in. executing gene expression, including bromodomain extra-terminal ((BET) (Borck et al., 2020). Some studies suggest that BET-containing family of epigenetic reader proteins, including BRD2, BRD3, BRD4 and the testis-restricted BRDT, provides a robust example of how epigenetic reader proteins can orchestrate transcriptional programs, provide new insight into mechanism of action and regulating effect and offer potentially novel therapeutic strategies in cardiovascular (Lin and Du, 2020; Li L. et al., 2021). Inhibition of BET epigenetic reader proteins might thus represent a promising therapeutic strategy to prevent adverse vascular remodelling (Dutzmann et al., 2021). BRD4, as a BET family member, plays an important role in critical biological processes. WU et al. found that BRD4 expression was up-regulated in human and mouse hypertrophied hearts, and importantly these effects were modulated by reactive oxygen species generation (Zhu W. et al., 2020). In one study, it has been reported that BETs are critical effectors of pathologic cardiac remodeling via their ability to co-activate defined stress-induced transcriptional programs in the heart (Auguste et al., 2020). Taken together, with the in-depth study of epigenetics, the secrets of related mechanisms will gradually be revealed.

The research on MI genetics contributes to early detection and the ability to provide personalised medical care. The MI pathophysiology would be progressively deciphered, demonstrating that genetics and epigenetics expedited MI onset and progression, enriching candidate methods (Nakatochi et al., 2017). Interactions between the genetic, epigenetic, and environmental factors constitute the critical factors of MI onset. The emergence of new genetic methods such as genome wide association analyses (GWAS) avoided some of these restrictions. GWAS analyses have exhibited that although different nationalities have different susceptibility genes and degrees of MI, multiple crucial loci have been identified for MI by GWAS (Takeuchi et al., 2012; Wakil et al., 2016). Epigenetic studies in cardiovascular medicine will improve our understanding of the molecular pathogenesis of MI and most importantly, facilitate novel biomarker identification, improved disease prevention, and new therapeutic strategies in managing MI. Future research should clarify how epigenetic mechanisms affect the MI process and prognosis to identify new drug targets and therapeutic strategies for MI. Although there is no specific drug for the epigenetic action of MI in clinic, currently available therapies, such as those using statins to promote epigenetic-based control in cardiovascular disease prevention through histone modifications, are already moving towards an exploitation of these mechanisms.
